# Identification of a Putative Network of Actin-Associated Cytoskeletal Proteins in Glomerular Podocytes Defined by Co-Purified mRNAs

**DOI:** 10.1371/journal.pone.0006491

**Published:** 2009-08-04

**Authors:** Behnam Nabet, Arthur Tsai, John W. Tobias, Russ P. Carstens

**Affiliations:** 1 Department of Medicine, University of Pennsylvania School of Medicine, Philadelphia, Pennsylvania, United States of America; 2 Department of Genetics, University of Pennsylvania School of Medicine, Philadelphia, Pennsylvania, United States of America; 3 Penn Bioinformatics Core, University of Pennsylvania School of Medicine, Philadelphia, Pennsylvania, United States of America; Lehigh University, United States of America

## Abstract

The glomerular podocyte is a highly specialized and polarized kidney cell type that contains major processes and foot processes that extend from the cell body. Foot processes from adjacent podocytes form interdigitations with those of adjacent cells, thereby creating an essential intercellular junctional domain of the renal filtration barrier known as the slit diaphragm. Interesting parallels have been drawn between the slit diaphragm and other sites of cell-cell contact by polarized cells. Notably mutations in several genes encoding proteins localized to the foot processes can lead to proteinuria and kidney failure. Mutations in the Wilm's tumor gene (*WT1*) can also lead to kidney disease and one isoform of WT1, WT1(+KTS), has been proposed to regulate gene expression post-transcriptionally. We originally sought to identify mRNAs associated with WT1(+KTS) through an RNA immunoprecipitation and microarray approach, hypothesizing that the proteins encoded by these mRNAs might be important for podocyte morphology and function. We identified a subset of mRNAs that were remarkably enriched for transcripts encoding actin-binding proteins and other cytoskeletal proteins including several that are localized at or near the slit diaphragm. Interestingly, these mRNAs included those of α-actinin-4 and non-muscle myosin IIA that are mutated in genetic forms of kidney disease. However, isolation of the mRNAs occurred independently of the expression of WT1, suggesting that the identified mRNAs were serendipitously co-purified on the basis of co-association in a common subcellular fraction. Mass spectroscopy revealed that other components of the actin cytoskeleton co-purified with these mRNAs, namely actin, tubulin, and elongation factor 1α. We propose that these mRNAs encode a number of proteins that comprise a highly specialized protein interactome underlying the slit diaphragm. Collectively, these gene products and their interactions may prove to be important for the structural integrity of the actin cytoskeleton in podocytes as well as other polarized cell types.

## Introduction

The renal glomerulus is the site of plasma ultrafiltration and disruption of glomerular function leads to progressive renal insufficiency and end-stage renal disease (ESRD). The glomerular filtration barrier is comprised of the fenestrated capillary endothelium, the glomerular basement membrane (GBM), and intercellular junctions between glomerular epithelial cells called podocytes, which overlie the GBM [1], [2]. The podocyte, a highly specialized kidney cell type consists of a cell body, major processes, and foot processes. The foot processes attach to the GBM and form extensive interdigitations with foot processes of adjacent podocytes to form a critical component of the filtration barrier termed the slit diaphragm. The slit diaphragm consists of several proteins that span the intercellular junctions with adjacent foot processes which prevent the passage of large molecules. Importantly, disruption of podocyte function that disrupts the slit diaphragm often leads to nephrotic syndrome and progressive deterioration in renal function [1], [2].

The complex structure of the podocyte is dependent upon a highly structured cytoskeleton that is critical for the maintenance of normal morphology and function. This highly dynamic network is involved in meeting the ongoing mechanical demands of filtration as well responses to cellular stress and injury [3]–[6]. Glomerular diseases that lead to proteinuria are associated with foot process effacement, which involves alterations in the actin cytoskeleton that retard normal slit diaphragm organization and function [3]–[6]. Thus, proteins that are components of the actin cytoskeleton, as well as proteins and pathways involved in its regulation, are crucial for its function.

Mutations in genes encoding proteins involved in slit diaphragm organization and actin cytoskeleton organization have been shown to play important roles in the progression of glomerular disease. Mutations in the gene encoding for nephrin, a transmembrane adhesion protein, has been shown to be the cause of nephrotic syndrome of the Finnish type [7]. In addition to nephrin, genetic mutations in components of the slit diaphragm including podocin (*NPHS2*), CD2-associated protein (*CD2AP*), and transient receptor potential cation channel 6 (*TRPC6*) have been identified in inherited nephrotic syndromes leading to ESRD [8]–[11]. Notably, TRPC6 has been shown to function in the regulation of calcium influx at the slit diaphragm and increased TRPC channel activity has been associated with reorganization of the actin cytoskeleton. Given that mutations associated with inherited nephrotic syndrome are characterized by increased channel activity, TRPC6 activity can be functionally linked to cytoskeletal regulation and maintenance [10], [11].

The importance of the actin cytoskeleton in glomerular and podocyte function is highlighted by mutations in α-actinin-4 (*ACTN4*), which leads to familial focal and segmental glomerulosclerosis (FSGS) [12]. Mutations in non-muscle myosin IIa (*Myh9*), lead to nephrotic syndrome in association with deafness, macrothrombocytopenia, and leukocyte inclusions in several syndromes such as Fechtner and Epstein syndromes, which might collectively be termed “Myh9 related diseases” [13]–[15]. The importance of Myh9 in podocytes is further illustrated by two recent studies which, using genome-wide association analysis through admixture linkage disequilibrium, identified multiple Myh9 SNPs that were associated with the increased risk of non-diabetic ESRD and FSGS in African American populations [16], [17].

In addition to the mutations described above, mutations in the Wilm's tumor gene (*WT1*) have also been associated with glomerular disease. *WT1* was originally identified as a gene mutated in patients with Wilm's nephroblastomas and mutations in *WT1* have been shown to be the cause of nephrotic syndrome. *WT1* mutations that lead to Denys-Drash syndrome and Frasier syndrome are each associated with nephrotic syndrome, renal failure, and intersex disorders, although only the former is commonly associated with Wilm's tumor [18]. Subsequent work has also shown that *WT1* is required for renal organogenesis and the development of other organ systems [19]–[21]. *WT1* encodes a protein containing four zinc finger domains whose expression in the mature kidney is limited to the podocyte. Alternative splicing of the WT1 transcript results in the inclusion or skipping of 9 nucleotides in exon 9 that encode the amino acids Lysine-Threonine-Serine (KTS) that are consequently present or absent between the third and fourth zinc finger domains of the protein. The protein isoform that does not contain the amino acids, WT1(-KTS) has been abundantly shown to function as a transcription factor. By contrast, the role of the more abundant protein isoform containing these amino acids, WT1(+KTS) has been less well defined, although it has been suggested that WT1(+KTS) functions as an RBP involved in post-transcriptional gene regulation [22], [23]. Evidence that the WT1(+KTS) isoform has important roles in podocyte function is provided by *WT1* mutations in patients with Frasier syndrome cause a reduction in relative levels of the WT1(+KTS) isoform. Similarly, mice with complete absence of WT1(+KTS) develop abnormal podocyte morphology and impaired foot process formation leading to renal failure [24].

Immunoprecipitation of RNA binding proteins and identification of the associated cellular RNAs by microarray analysis (often called “RIP-CHIP”) has been successfully used by a number of groups (Reviewed in [25]). To investigate the potential post-transcriptional regulatory functions of WT1(+KTS) we developed a variation to the RIP-CHIP protocol. We sought to identify a subset of WT1(+KTS)-associated mRNAs in podocytes that might be physiologically important for glomerular function. In order to identify potential candidate mRNAs associating with WT1(+KTS), WT1(+KTS) was purified from cell extracts from a podocyte cell line stably expressing an epitope tagged WT1(+KTS) construct. DNA microarrays were then used to identify candidate target mRNAs. An underlying hypothesis was that the proteins encoded by mRNAs bound by WT1(+KTS) might define a “post-transcriptional operon” that is important for maintenance of podocyte morphology and function [26]. According to this hypothesis, RBPs with important cellular functions co-regulate the expression of gene transcripts that function in common biochemical pathways or are present in common macromolecular structures. The mRNAs that were co-purified in this study demonstrated a remarkable enrichment for transcripts encoding components of a protein-protein interaction network that comprises the actin cytoskeleton. However, these mRNAs were subsequently, and unexpectedly, shown not to be bound by WT1(+KTS). However, while our findings did not address the original hypothesis that WT1(+KTS) directly binds mRNAs *in vivo*, we nonetheless identified transcripts that encode a subset of interrelated, physiologically relevant proteins that we propose will provide further insights into actin cytoskeletal organization.

## Results

### A modified RIP-CHIP approach used to identify functionally relevant mRNAs

**Figure 1:**
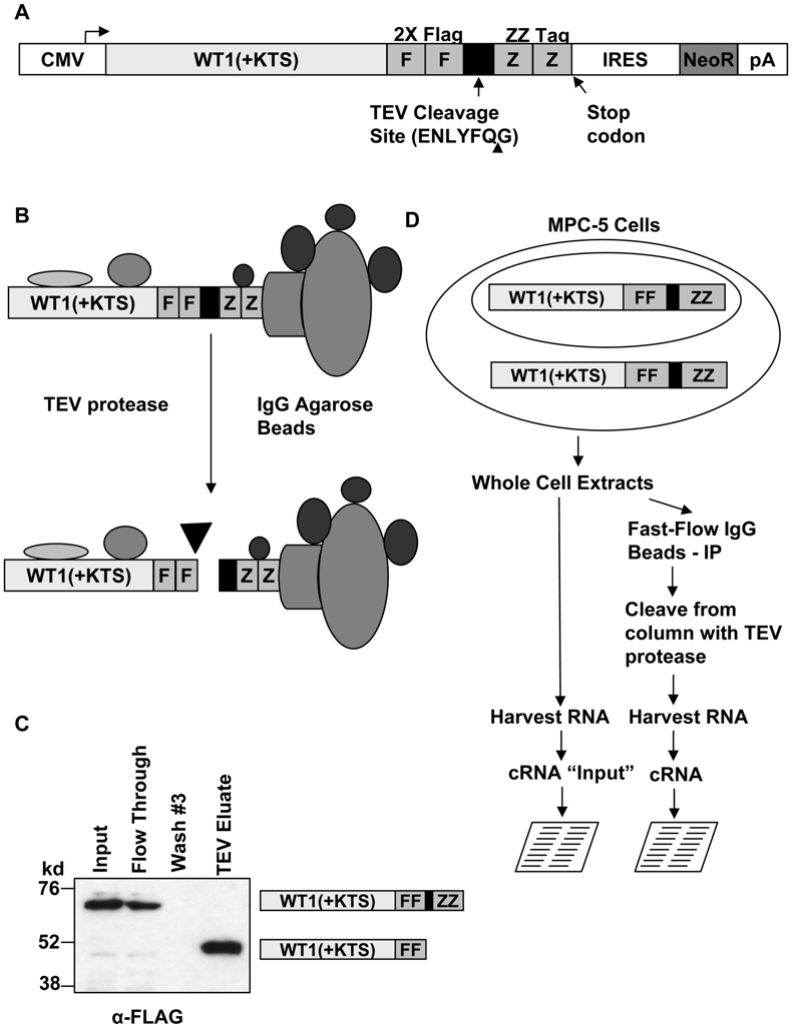
Schematic of RIP-CHIP approach used to identify functionally relevant mRNAs. (A) Diagram of the cDNA expression vector containing two FLAG tags (“FF”) and two copies of the IgG binding domain of protein A (“ZZ”). The tobacco etch virus (TEV) protease cleavage site is indicated, including an arrowhead to designate cleavage between glutamine and glycine residues. CMV, cytomegalovirus promoter; NeoR, neomycin resistance cassette; pA, polyadenylation site. (B) Schematic of purification strategy using TEV protease cleavage. ZZ-tagged protein is bound to IgG beads, washed, and the protein, along with co-purified mRNAs, are eluted from the beads by TEV protease cleavage. (C) Demonstration of WT1(+KTS)-FF-ZZ expression in differentiated MPC-5 cells (“Input”) and the elution of WT1(+KTS)-FF bound to the beads after 3 washes and TEV protease cleavage as determined by immunoblotting with anti-FLAG antibodies. (D) Flowchart summarizing the RIP-CHIP approach.

To investigate the potential post-transcriptional role and target transcripts of WT1(+KTS) in podocytes, we developed a modified RIP-CHIP approach using an expression construct originally developed for a tandem affinity purification (TAP) protocol [27]. This expression construct contains a tag (“FF-ZZ”) consisting of two copies of a C-terminal FLAG tags (“FF”) separated from two more C-terminal IgG binding domains (“ZZ”) of protein A by a cleavage site for the site specific tobacco etch virus (TEV) protease (Figure 1A) [27], [28].

In order to identify gene transcripts encoding podocyte-relevant products, we made use of the conditionally immortalized mouse MPC-5 podocyte cell line [29]. MPC-5 cells were established with stable expression of the WT1(+KTS) cDNA containing the FF-ZZ tag. While no cell line is completely representative of any primary cell type *in vivo*, the MPC-5 cell line expresses most known podocyte cell markers and has been extensively validated in numerous studies to provide insights into podocyte function and glomerular disease pathogenesis [30]. The MPC-5 cells express a temperature sensitive, interferon driven SV40 T antigen construct that facilitates propagation at 33°C. Upon transfer to 37°C and withdrawal of interferon, SV40 expression is turned off and existing T antigen is inactivated whereupon the cells cease proliferation and differentiate over 6–14 days [29].

Cell extracts were prepared from WT1(+KTS)-FF-ZZ expressing MPC-5 cells that were allowed to differentiate at 37°C for 14 days. Extracts were then incubated with IgG Sepharose Fastflow beads, washed, and eluted from the column by cleavage with TEV protease (Figure 1B) [27]. A Western blot demonstrating successful cleavage by TEV protease is shown in Figure 1C. As a control, RNA was prepared from the input extracts that were incubated without IgG beads for the same amount of time as the experimental samples that were bound to the IgG beads. Biotin-labelled cRNAs for microarray analysis were prepared from the input and eluate samples and microarray analysis was conducted using Illumina Sentrix 6 mouse Beadchips (Figure 1D).

### Identification of reproducibly enriched mRNA transcripts in pulldowns from MPC-5 cell extracts

To achieve a statistically robust dataset, four independent RNP pulldowns with the IgG beads were completed. Subsequently, four independent pairwise comparisons between these RNP pulldowns and the input samples processed in parallel with each experimental pulldown were carried out. To analyze the probesets that passed the expression filter, we used a 15% False Discovery Rate (FDR) cutoff and an average 2-fold enrichment cutoff. Using the 15% FDR cutoff, this analysis yielded a list of 88 probesets corresponding to enrichment of 60 unique genes (Table 1, Table S1). Notably, a number of mRNAs displayed a significantly increased fold-enrichment compared to the input samples.

**Table 1:** Co-purified mRNAs enriched in eluates relative to input extracts.

Rank	Fold Enrichment	Symbol	Gene Name
1,10	14.2	Myh10	Non-muscle myosin IIb
2,11	12.9	Flnb	Filamin B
3,50	12.55	Dbn1	Drebrin
4,9,28,86	12.4	Svil	Supervillin
5,40	11.14	Ppp1r9a	Neurabin I
6,13,36	10.61	Spnb2	Spectrin β2
7,50	10.33	Utrn	Utrophin
8,19	10.06	Plec1	Plectin
12,65	9.14	2900026A02Rik	Riken cDNA 2900026A02 gene
14	8.04	Cgn	Cingulin
15,16	7.42	Lima1	Eplin/Lim domain and actin binding 1
17	6.44	St5	Supp. of tumorigenicity 5
18,37	6.24	Cgnl1	Cingulin-like
20	6.05	Aim1	Absent in Melanoma 1
21	5.79	Flna	Filamin A
22	5.54	Myo6	Myosin VI
23,24,29	5.46	Actn4	α-Actinin-4
25	4.89	Nes	Nestin
26,31	4.86	Iqgap1	IG motif GTPase activating protein
27	4.85	Fbxo34	F-box only protein 34
30	4.27	Specc1	Sperm antigen with calponin homology and coiled-coil domains 1
32	4	C430004E15Rik	Riken cDNA C430004E15 gene
33	3.87	Mical3	Microtubule associated monoxygenase, calponin and LIM domain 3
34,39	3.85	Spna2	Spectrin α2/α-Fodrin
35,46,47,55	3.8	Dst	Dystonin
38	3.71	Myo1e	Myosin 1e
41,83	3.63	Actn1	α-Actinin-1
42	3.54	Myo18a	Myosin XVIIIa
43	3.33	Flnc	Filamin C
44	3.29	Myh9	Non-muscle myosin IIa
45	3.27	Eppk1	Epiplakin
48	2.93	Dmn	Desmuslin
49,59	2.83	Lmo7	Lim domain only 7
51,57	2.7	Myo5a	Myosin heavy chain 12
52	2.64	Ppp1r9b	Neurabin II/Spinophilin
53	2.64	Fbxo46	F-box only 46
54	2.63	cep350	Centrosome associated protein 350
58	2.53	Pls3	T-Plastin/T-Fimbrin
60,70	2.5	Smtn	Smoothelin
61	2.45	Zfp185	Zinc finger protein 185
62	2.42	Myo1b	Myosin Ib
63,66	2.4	Gas2l3	Growth arrest specific 2-like 3
64	2.37	Crocc	Rootletin
67	2.29	Shrm	Shroom
68	2.29	Incenp	Inner centromere protein
69,75	2.27	Baz1a	Bromodomain adjacent to zinc finger domain 1A
71	2.22	Myo1d	Myosin 1d
72	2.22	Supt6h	Suppressor of Ty 6 homolog
73	2.21	2610015P09Rik	Riken cDNA 2610015P09 gene
74	2.2	Gsn	Gelsolin
77	2.2	Dnmt1	DNA methyltransferase 1
78	2.18	Ppp1r8	Protein phosphatase 1 regulatory inhibitor subunit 8
79	2.17	Cspp1	Centrosome and spindle pole associated protein
80	2.14	Crybb3	Crystallin B3
81	2.14	Zc3h13	Zinc finger CCCH-type containing 13
82	2.14	Pprc1	Peroxisome proliferator-activated receptor gamma, coactivator-related 1
84	2.05	Arhgap23	Rho GTPase activating protein
85	2.03	Ogdh	Oxoglutarate dehydrogenase (lipoamide)
87	2.03	Mcm7	Minichromosome maintenance deficient
88	2.01	Tmod3	Tropomodulin 3

List of genes that passed the 15% FDR cutoff and were more than two-fold enriched. The rank column also lists genes with multiple corresponding probesets. For genes with multiple probesets in the list, the fold change for the highest ranked probeset is shown. For the complete list of all genes with the corresponding fold changes see Table S1.

**Figure 2:**
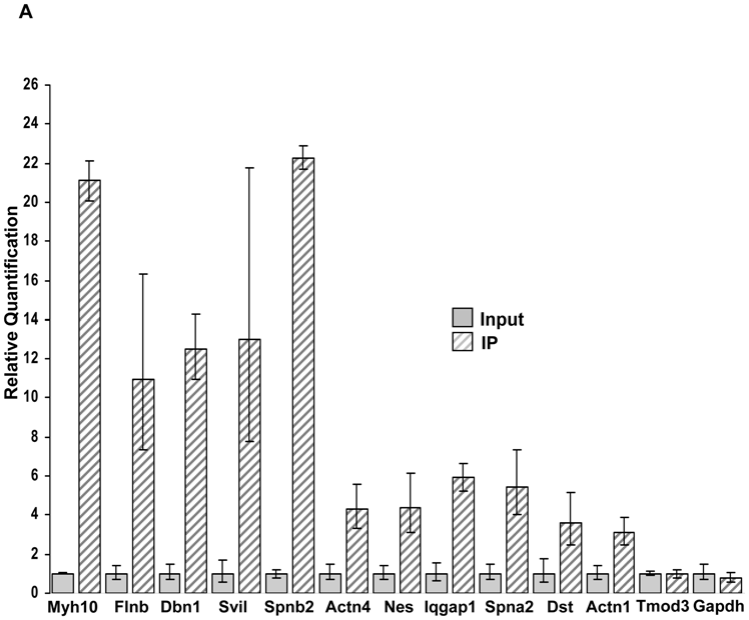
Enriched mRNAs identified by microarray analysis are independently validated by qRT-PCR. Quantitative RT-PCR analysis of a subset of the enriched mRNAs identified in this study. Two independent pulldowns using MPC-5 cell extracts stably WT1(+KTS)-FF-ZZ were completed and total RNAs from input extracts and WT1(+KTS) enriched fractions following TEV protease cleavage were compared. Mean expression values from triplicate assays±SD are shown relative to the input control samples.

Quantitative real-time RT-PCR (qRT-PCR) was used to independently validate enrichment of 12 of the enriched mRNAs with variable levels of enrichment in the microarray results. For this validation two independent pulldowns were performed using fully differentiated WT1(+KTS) expressing MPC-5 cells. Analysis was completed for each mRNA in the pulldown as compared to the levels in control total input extracts. Enrichment of 11 of the 12 putative transcripts was validated with fold-enrichment that was approximately similar to that observed using microarrays (Figure 2). Notably, the one transcript for which qRT-PCR did not demonstrate enrichment in the pulldown, *Tmod3*, represented the least enriched gene transcript for which a probeset on the microarray showed at least a two-fold enrichment. Thus, taken together, the qRT-PCR data suggested that the enrichment of the transcripts identified by microarray analysis was reproducible. Notably, among the genes with the highest level of fold-enrichment we frequently observed more than one probeset corresponding to the same transcript, thereby providing additional evidence of the validity and reproducibility of the microarray results.

### Transcripts identified by RIP-CHIP analysis are highly enriched for those that encode components of the actin cytoskeleton

To characterize the functions and interactions of the proteins encoded by mRNAs from this list, the top 60 genes which passed the 15% FDR cut-off and were more than 2-fold enriched were compared to the background set of the all genes in the mouse genome using Gene Ontogeny (GO) analysis through the DAVID Bioinformatics Database (Table 2) [31], [32]. Most notable was a clearly defined enrichment in proteins encoded by these transcripts that were components of the cytoskeleton (60.0%; p-value: 2.0×10^−29^), were involved in cytoskeletal organization (34.5%; p-value: 2.2×10^−16^), and were actin binding (45.5%; p-value: 4.7×10^−31^).

**Table 2:** Gene Ontogeny (GO) Analysis.

Category	GO term	#Of Genes (%)	p-value	Benjamini-Hochberg
Molecular Function	Actin binding	25 (45.5)	4.7×10^−31^	1.3×10^−27^
	Cytoskeletal protein binding	27 (49.1)	1.4×10^−30^	1.8×10^−27^
	Calmodulin binding	10 (18.2)	2.3×10^−11^	2.1×10^−8^
	Motor Activity	10 (18.2)	3.9×10^−10^	2.7×10^−7^
Cellular Component	Cytoskeleton	33 (60.0)	2.0×10^−29^	1.6 ×10^−26^
	Actin cytoskeleton	21 (38.2)	6.8×10^−26^	2.7×10^−23^
	Non-membrane bound organelle	35 (63.6)	4.4×10^−25^	1.1×10^−22^
	Cytoskeletal part	20 (36.4)	4.8×10^−16^	7.0×10^−14^
	Myosin complex	10 (18.2)	1.1×10^−13^	1.4×10^−11^
Biological Processes	Cytoskeletal organization	19 (34.5)	2.2×10^−16^	1.2×10^−12^
	Actin filament-based process	14 (25.5)	2.8×10^−15^	7.2×10^−12^
	Actin cytoskeleton organization	12 (21.8)	1.8×10^−12^	3.1×10^−9^

List of the most statistically significant GO terms resulting from analysis between enriched genes and the entire mouse genome background set. The number and percentage of genes in each category are provided. The p-value (EASE score) indicates the probability that these genes are enriched in these categories due to random chance. The Benjamini-Hochberg score globally corrects enrichment p-values to account for multiple testing.

This analysis, together with the readily apparent number of actin binding proteins among the list prompted further investigation into the shared domains and potential interactions among the proteins encoded by the enriched mRNAs. The most common protein domains are listed in Table 3. We noted that the most enriched motif among these proteins (present in 17 proteins) was the actin-binding calponin homology (CH) domain. In 10 of these proteins, this was the actinin-type subtype of CH domain. Given the number of myosin proteins, there was also an expected enrichment for domains common to the myosin family of proteins, including the IQ calmodulin-binding motif and myosin head and tail domains. In addition to the alpha-actinins and myosins, there are several other examples in which more than one member of a family of proteins was present in the list, or in which homologous proteins were present. This included identifying all three of the filamins (Flna, Flnb, and Flnc), non-erythrocytic spectrins α2 and β2 (Spna2 and Spnb2; also called α and β fodrin), cingulin and cingulin-like (Cgn and Cgnl), neurabin I and neurabin II (Ppp1r9a and Ppp1r9b), and three members of the plakin family of proteins: plectin, dystonin, and epiplakin (Plec, Dst, and Eppk1). In addition to the myosins and proteins with CH actin-binding domains, there were a number of additional proteins on the list of proteins that are known to be actin-binding proteins and for which a direct interaction with actin has been shown in the published literature (Table 4). In fact, over half of the list genes encoded actin-binding proteins (33/61) and several more, spectrin α2 (Spna2), cingulin-like (Cgnl), and phostensin (Kiaa1949) have been shown to co-localize with actin and/or are implicated in actin binding. These results therefore provided further evidence that the co-purified mRNAs encode numerous proteins that are important components of the actin-cytoskeleton.

**Table 3:** Protein domains enriched in products of identified mRNAs.

Common Domains	Proteins Encoded by Enriched mRNAs
Calponin homology domain	Flnb, Spnb2, Utrn, Plec1, Flna, Actn4, Iqgap1, Mical3, Specc1, Dst, Actn1, Flnc, Pls3, Lmo7, Smtn, Gas2l3, Mical3
Actin-binding, actinin-type	Flnb, Spnb2, Utrn, Plec1, Flna, Actn4, Dst, Actn1, Flnc, Pls3
Tropomyosin	Myh10, Ppp1r9a, Cgn, Cgnl, Specc1, Myo18a, Myh9, Myo1b, Crocc, Incenp, Ppp1r9b
IQ calmodulin-binding motif	Myh10, Myo6, Iqgap1, Myo18a, Myh9, Myo1b, Myo1e, Myo5a, Myo1d
Spectrin repeat	Spnb2, Utrn, Plec1, Actn4, Spna2, Dst, Actn1
ATP/GTP-binding site motif A (P-loop)	Myh10, Myo6, Dst, Myh9, Myo1b, Gas2l3
Myosin tail	Myh10, Cgn, Cgnl, Myh9, Myo1b, Myo1e, Myo1d
Myosin head	Myh10, Myo6, Myo18a, Myh9, Myo1b, Myo1e, Myo5a, Myo1d
Calcium binding EF Hand	Actn4, Spna2, Dst, Actn1, Pls3
Proline-rich region	Dbn1, C430004e15Rik, BC030863, Smtn, 2310014H01Rik, Shrm
LIM zinc-binding domain	Lima1, Mical3, Lmo7, Mical3, Zfp185
PDZ domain	Ppp1r9a, Myo18a, Lmo7, Ppp1r9b, Shrm
Filamin/ABP280 repeat	Flnb, Flna, Flnc
Prefoldin	Cgnl, Specc1, Crocc
Ig-like fold	Flnb, Flnc
WW/Rsp5/WWP	Utrn, Iqgap1
Beta and Gamma crystalline	Aim1, Crybb3
GAS2 domain	Dst, Gas2l3
Pleckstrin-like	Spnb2, Arghap23
Plectin repeat	Plec1, Dst, Eppk1
SH3 domain	Spna2, Dst, Myo1e

**Table 4:** Enriched mRNAs that encode proteins with well established and/or have been shown to bind directly to actin.

Gene Symbol	Actin Binding domain	Reference
Myh10	Myosin head	
Flnb	Alponin homology domain/actinin-type actin-binding domain	
Dbn1	Cofilin/tropomyosin type actin-binding domain	[72]
Svil	Gelsolin homology domain	[73]
Ppp1r9a	neural F-actin binding protein	[74], [75]
Spnb2	Calponin homology domain/actinin-type actin-binding domain	
Utrn	Calponin homology domain/actinin-type actin-binding domain	[76], [77]
Plec1	Calponin homology domain/actinin-type actin-binding domain	[78]
Cgn		[79]
Lima1		[80]
Flna	Calponin homology domain/actinin-type actin-binding domain	
Myo6	Myosin head	
Actn4	Calponin homology domain/actinin-type actin-binding domain	
Iqgap1	Calponin homology domain	[81]
Specc1	Calponin homology domain	
Mical3	Calponin homology domain	
Dst	Calponin homology domain/actinin-type actin-binding domain	
Myo1e	Myosin head	
Actn1	Calponin homology domain/actinin-type actin-binding domain	
Myo18a	Myosin head	[82]
Flnc	Calponin homology domain/actinin-type actin-binding domain	
Myh9	Myosin head	
Ppp1r9b		[83], [84]
Pls3	Calponin homology domain/actinin-type actin-binding domain	[85]
Lmo7	Calponin homology domain	[86]
Myo5a	Myosin head	
Smtn	Calponin homology domain	[87]
Myo1b	Myosin head	
Gas2l3	Calponin homology domain	[88]
Shrm	APX/Shrm domain 1	[89]
Myo1d	Myosin head	
Gsn	Gelsolin homology domain	
Tmod3		[90]

### Co-purified mRNAs encode proteins that are important components of podocyte foot processes, including gene products implicated in glomerular disease

A number of the proteins encoded by the identified mRNAs have been directly linked to functions that are critical for the maintenance of the structural integrity of the podocyte and in the process of glomerular filtration. Eight of encoded proteins have been directly shown to localize to the podocyte foot processes, including the slit diaphragm: myosin IIa (Myh9), myosin IIb (Myh10), drebrin (Dbn1), spectrin-α2 (Spna2), spectrin-β2 (Spnb2), α-actinin-1 (Actn1), α-actinin-4 (Actn4), and utrophin (Utrn) [12], [15], [33]–[37]. As previously discussed, *Myh9* and *Actn4* encode proteins that are predominately expressed in the podocyte within the glomerulus and have been associated with genetic forms of glomerular disease. Interestingly, biochemical purification of Actn4 identified Myh9 as the predominant interacting protein, suggesting that interactions between these proteins are a part of a protein interaction network in podocytes that underlie maintenance of structural cytoskeletal integrity [38]. Notably in the list of genes that passed the 15% FDR, three positive probesets for *Actn4* were present in the top 30 ranked probesets, providing strong evidence of reproducible enrichment.

Nephrin is one of the most important proteins for maintenance of slit diaphragm structure and function. Numerous studies have shown that connections between the cytoplasmic tail of nephrin to the underlying actin cytoskeleton are critical for its function [39]. While the Nephrin mRNA was not detected in our podocyte cell line extracts by the single probeset corresponding to it, a number of nephrin-interacting proteins were identified in this study. For example, it has been suggested that IQGAP1, SPNA2, SPNB2, and actinin proteins form a “nephrin multiprotein complex” which are important components structurally and functionally linking nephrin to the actin cytoskeleton [35], [40]. Interestingly, IQGAP1, an important cytoskeletal adhesion protein, not only colocalizes with Nephrin but also has been shown to interact with Phospholipase C epsilon (PLCE1). *PLCE1* mutations have been shown to result in familial nephrotic syndrome suggesting that such interactions may be crucial in cell adhesion and reorganization as well as maintaining the structural integrity of the actin cytoskeleton [34].

As has been shown for *ACTN4*, mutations in *TRPC6* are also associated with familial FSGS. TRPC6 has also been localized to the slit diaphragm and a recent study identified proteins associated with TRPC5 and TRPC6 [41]. Using antibodies for TRPC5, this study listed 15 unique cytoskeletal proteins of which 11 were encoded by our target mRNAs: Spna2, Spnb2, Myh9, Myh10, Myo5a, Myo6, Actn1, Actn4, Dbn1, Pppar9a, and Ppp1r9b. While not all of these proteins were specifically shown to be TRPC6 binding proteins in this publication, one of the authors reports that at least eight, Spna2, Spnb2, Myh9, Myh10, Actn1, Acnt4, Dbn1, Gsn, do also specifically co-IP with TRPC6 (W.P. Schilling, personal communication). It has been suggested that TRPC6 functions at the slit diaphragm to transmit Ca flux into signaling pathways that are involved in reorganization of the actin cytoskeleton [42], [43]. Thus, these proteins that associate in complexes with TRPC6 may likewise comprise important components of the foot process cytoskeleton.

Abnormal expression levels of several of the enriched mRNAs have also been observed in various renal disease models. In a mouse model of HIV associated nephropathy (HIVAN), a reduction in the expression of filamin A (Flna) was seen in podocytes [44]. The authors suggested that reduced filamin expression was likely one factor that led to cytoskeletal abnormalities, or “softness” in HIVAN podocytes compared to wild-type podocytes. The mRNAs for *Flna*, *Flnb*, and *Flnc* were all enriched in our study, indicating that maintenance of their expression in response to stress thus might be one potential functional role of this isoform. By contrast, another of our highly enriched mRNAs, that encoding myosin IIb (*Myh10*) has been shown to be overexpressed in a number of glomerular disease models (e.g. [45]). Thus, collectively, review of the literature reveals that a number of the proteins encoded by the mRNAs we identified are implicated in critical functions of the podocyte and thus, by extension, may potentially be involved in glomerular pathogenesis as well.

### Enrichment of co-purified mRNAs was not WT1(+KTS) dependent

To date α-actinin-1 (*Actn1*) is the only identified cellular mRNA shown to be bound by WT1(+KTS) [46]. Two positive probesets for *Actn1* were present in the top 88 ranked probesets. This observation was consistent with the hypothesis that the mRNAs that we identified were bound by and associated specifically with WT1(+KTS). However, we were surprised to find that in subsequent validations, we detemined that the enrichment these mRNAs was clearly not due to association with WT1(+KTS).

**Figure 3:**
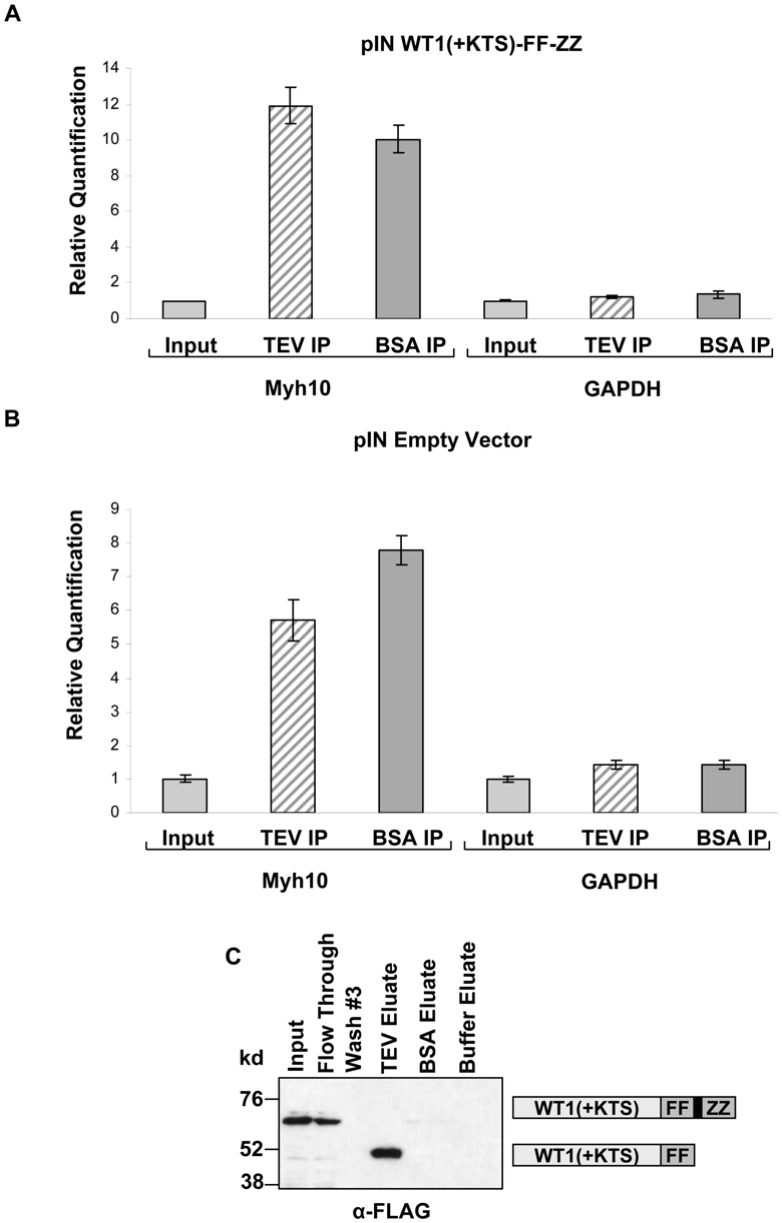
The enrichment of the mRNAs in the pulldown assays does not require association with WT1(+KTS). (A) Elution of Myh10 mRNA from WT1(+KTS)-FF-ZZ containing IgG beads occurs with addition of bovine serum albumen (BSA) as well as TEV protease. Shown is qRT-PCR analysis of Myh10 and Gapdh in IP's using cell extracts from MPC-5 cells stably expressing WT1(+KTS)-FF-ZZ. Mean expression values from triplicate assays±SD are shown relative to Input samples. (B) The same enrichment of Myh10 mRNA shown in (A) in response to TEV protease and BSA using extracts from cells expressing the pIN empty vector. Mean expression values from triplicate assays±SD are shown relative to Input samples. (C) Immunoblot demonstrating that WT1(+KTS)-FF-ZZ elution occurs only upon addition of TEV protease and not with addition of BSA or TEV cleavage buffer alone. Presence of the FLAG tagged WT1(+KTS) protein was determined using anti-FLAG antibodies.

Further validation experiments using qRT-PCR were carried out to more definitively determine whether the enrichment of these mRNAs was specifically due to association with WT1(+KTS). Additional pulldowns were performed, using cell extracts from MPC-5 cells stably expressing WT1(+KTS)-FF-ZZ, but also using control cell extracts from MPC-5 cells expressing the empty vector plasmid. Because of the significant enrichment of *Myh10*, we used qRT-PCR analysis of *Myh10* transcripts for these validation steps. To our initial surprise, we noted a significant enrichment of *Myh10* mRNA in the eluates of both the experimental (Figure 3A) and control samples (Figure 3B). These results were highly reproducible and thus the enrichment of *Myh10* in the pulldowns using empty vector control extracts clearly indicated that the enrichment of this mRNA did not require an association with WT1(+KTS). WT1(+KTS) independent enrichment of a number of the other candidate mRNAs was also confirmed by qRT-PCR in the empty vector pulldowns, including *Dbn1* (data not shown). Thus, despite the identification of a previously described WT1(+KTS) target mRNA in the results and the remarkable physiologically relevant functions of the encoded proteins, these results established that their identification was not due to WT1(+KTS) binding as envisioned by the experimental design.

### Protein components of the actin cytoskeleton were co-purified with the enriched mRNAs

Although the co-purified mRNAs were not enriched due to an association with WT1(+KTS), the significant examples of their common functions in the actin cytoskeleton raised the question as to how or why they were specifically enriched in the pull down assays. Given the functional interrelationships among the set of identified transcripts, we therefore sought to determine the basis on which purification approach unveiled this subset of enriched mRNAs.

**Figure 4:**
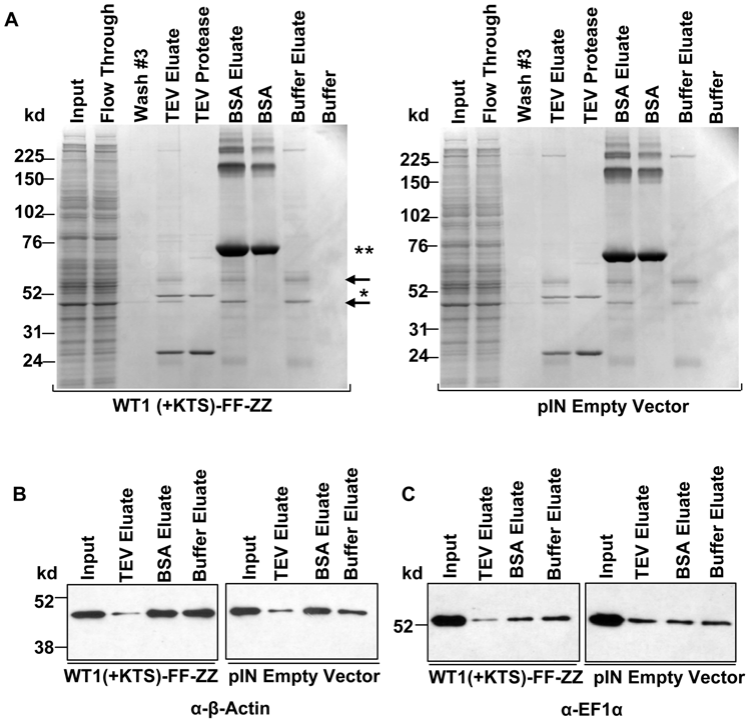
Protein components of the actin cytoskeleton are present in eluates used to identify the enriched subset of mRNAs. (A) Coomassie stained gels demonstrating proteins present in the IP eluates using MPC-5 cell extracts stably expressing WT1(+KTS)-FF-ZZ or empty vector expression constructs. Arrows correspond to the bands from TEV eluate lane of the WT1(+KTS)-FF-ZZ gel that were excised and submitted for mass spectrometry analysis. The same arrows also indicate the bands which correspond to tubulin and elongation factor 1α (top arrow) and actin (bottom arrow). * indicates TEV protease and ** indicates BSA bands. The actin (B) and elongation factor 1α (C) proteins are not enriched in the eluates from IgG beads. Immunoblots confirming the presence, but lack of enrichment of β-actin and elongation factor 1α in eluate samples from WT1(+KTS)-FF-ZZ and empty vector control pulldowns with anti-β-actin and anti-elongation factor 1α antibodies.

We initially hypothesized that the common enrichment of these mRNAs was due to an association with other RBPs, protein complexes, or a soluble protein fraction in the purified eluate samples. Therefore mass spectrometry was used to identify proteins in the purified eluates following TEV protease cleavage. It should be noted that this analysis was completed from bands that were excised from the WT1(+KTS) gel pictured in Figure 4A. This analysis revealed a predominant presence of actin, tubulin, and elongation factor 1α (EF1α) in the purified eluates. Alpha and gamma subunits of actin were identified corresponding to a total of 98 peptide matches of the protein sequence, beta and alpha tubulin subunits were identified corresponding to a total of 526 peptide matches of the protein sequence, and EF1α was identified corresponding to 52 peptide matches of the protein sequence.

The presence of actin and tubulin in the eluate fractions, suggested that a fraction containing cytoskeletal structural proteins may in part have accounted for our findings. However, it was not clear how these proteins might have been eluted following TEV protease cleavage given the specificity of this protease. We thus investigated whether the cleavage step of the ‘ZZ-tag’ by the TEV protease was in fact required for enrichment of these proteins and mRNAs. In order to investigate the role of the TEV protease, three separate pulldowns were completed using cell extracts from MPC-5 cells stably expressing WT1(+KTS). As an additional control, cell extracts from cells stably expressing the empty expression vector were used. In these pulldowns, MPC-5 cell extracts were incubated with IgG Sepharose Fastflow beads, washed, and eluted either by adding TEV protease to the column to promote cleavage of the ZZ-tag, an equal amount of BSA in place of TEV protease, or buffer alone to the column. Coomassie staining comparing each of these eluates revealed that, other than the bands corresponding to TEV protease or BSA bands, the eluates in all three cases revealed essentially the same set of protein bands (Figure 4A). Furthermore, the same bands were also seen in eluates from the empty vector control extracts. These bands represent those submitted for mass spectrometry analysis and therefore their presence in the eluates was not only independent of WT1(+KTS), but also did not specifically require elution with TEV protease (Figure 4A). The presence of β-actin and EF1α were also confirmed by Western blotting (Figure 4B and C). qRT-PCR analysis of these pulldowns also demonstrated that enrichment of *Myh10* mRNA was similarly observed in eluate samples using TEV protease or the BSA (Figure 3A and B). Additional Western blots confirmed that FLAG-tagged WT1 was still present in the TEV protease eluates following cleavage by TEV protease but, as expected, was not present in control pulldowns using BSA or buffer alone, providing definitive evidence that WT1 was not required for mRNA enrichment (Figure 3C).

Collectively, these data demonstrate that TEV protease cleavage and the presence of WT1(+KTS) was not required for identification of these mRNAs. The most straightforward technical detail to account for the presence of these proteins and mRNAs is based on the method by which washes and the elutions were carried out by spin column chromatography. The flow through and wash fractions were harvested by pelleting of the beads by centrifugation and removal of the respective supernatant fractions. While the final wash fractions were reproducibly clear of significant amounts of protein, the subsequent elutions were then performed using microbiospin columns. At this step then, the beads were trapped in the column and the eluates harvested from the material that was centrifuged through the filter contained residual protein material that previously was trapped with the beads. This data suggests that in these experiments the actin, tubulin, and EF1α were trapped by the IgG Sepharose Fastflow beads at each wash step and during the final elution, a semi-soluble fraction was forced through the column. Importantly, Western blots comparing input and purified eluate samples probing for Myh10, Dbn1, and IQGAP1 demonstrated little or no protein present in the purified eluate samples, compared to strong signals in the input samples (data not shown). This suggests that identification of these transcripts did not result from trapping of the cytoskeletal proteins that were encoded by the respective mRNAs. Rather, the protein fraction obtained likely represented a cytoplasmic complex of proteins in which the mRNAs identified by microarray analysis were associated and subsequently enriched relative to the input fractions.

In an attempt to further account for the co-enrichment of these mRNAs we also used a number of bioinformatics algorithms to investigate whether a common sequence motif could be identified in these transcripts. This analysis included the 3′ untranslated regions (UTRs) as well a 5′ UTRs and coding sequences. However, we were unable to identify a sequence motif that was common to most of these mRNAs and we did not identify significant enrichment for the binding sites of known RNA binding proteins (data not shown). These findings thus cannot establish whether these co-regulated mRNAs are bound by a single trans-acting factor, but suggest that more than one RNA binding protein is involved in their regulation. However, we suspect that the co-enrichment of these mRNAs in the purifications itself was not dependent upon direct binding to one or more of these trans-factors.

## Discussion

**Figure 5:**
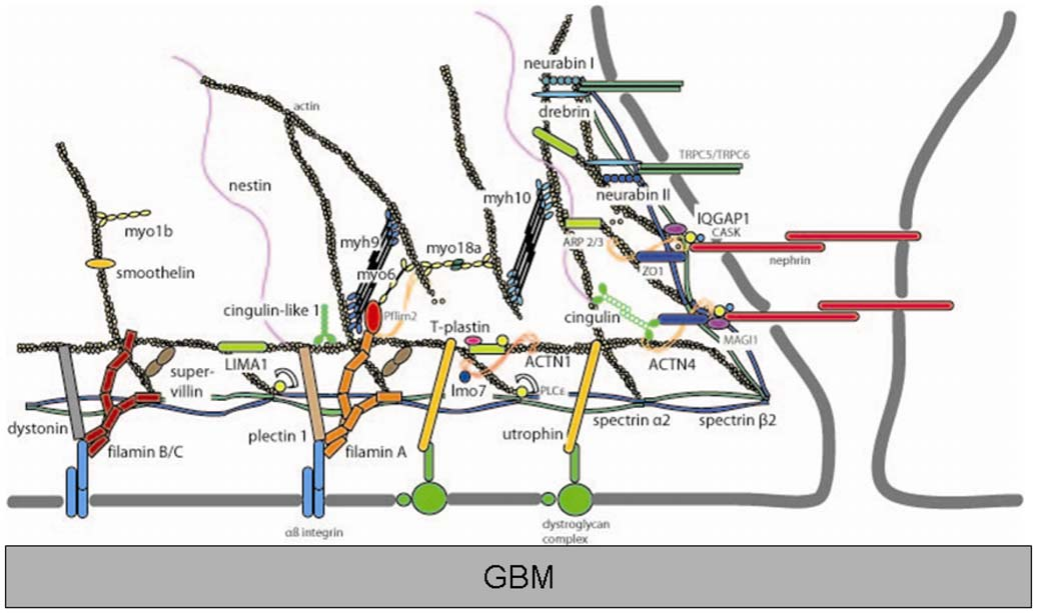
Schematic representation of a hypothesized podocyte protein “interactome” encoded by enriched mRNAs. Large black type indicates proteins encoded by mRNAs identified in this study. Smaller grey type is used to indicate several known podocyte foot process proteins in order to orient the figure. The interactions shown represent published results showing either direct interactions or presence in common protein complexes.

The structural and functional integrity of the podocyte is essential for the maintenance of the glomerular filtration barrier and components of the actin cytoskeleton have been shown to be crucial for podocyte function and response to stress and injury. However, it has also been noted that the podocyte cytoskeleton remains to be fully defined and thus it is likely that numerous undiscovered components are also important for podocyte function [44]. While our experiments were designed to uncover WT1(+KTS) associated mRNAs, we nonetheless identified through serendipity a subset of mRNA transcripts that encode for proteins that exhibit a remarkable degree of interactions with one another as components of the actin cytoskeleton. Extensive literature reveals that there are numerous instances in which these encoded proteins have been shown to interact with one another and/or to be present in common protein complexes in podocytes or other polarized cell types. Considering the collective set of interactions among these proteins and other well characterized podocyte proteins, a putative cytoskeletal “interactome” comprising these proteins can be envisioned (Figure 5). The protein interactions shown in this diagram represent known interactions that constitute important components of the actin cytoskeleton. However, numerous proteins with less well described functions that were identified in our analysis may also be shown to contribute important functions to this interactome. Thus, together, the collective links between these proteins suggests that they contribute to the structure and function of podocytes and to the reorganization of the cytoskeleton that occurs in response to the normal stresses involved in sustaining filtration function as well as in response to injury. Importantly, the genes that encode several of these mRNAs (e.g. *Actn4, Myh9*) have previously been implicated in genetic forms of kidney disease or the encoded proteins have been shown to interact with gene products that have been shown to play a role in glomerular disease. Consequently, given these direct and indirect links between the proteins and genetic glomerular diseases, we suggest that the genes we identified might be considered candidate genes whose mutation may also lead to glomerular disease due to podocyte pathophysiology. Furthermore, the proteins, pathways and structural interactions that are defined by these mRNAs may also be involved in the pathophysiology of secondary causes of the nephritic syndrome that may interfere with their function through alternative mechanisms. In sum, we hypothesize that the proteins encoded by these mRNAs include a number of previously uncharacterized podocyte proteins that might play essential roles in maintaining the morphology and function of this highly specialized cell type. It is hoped that the importance of the defined proteins, including previously uncharacterized proteins, and their interactions in the podocyte will be better clarified through future cellular and molecular investigations.

The proteins encoded by these mRNAs may not only play an important role in maintaining the structure of the podocyte actin cytoskeleton, but also function in regulating the actin cytoskeleton in other polarized cell types. Structural and functional parallels have been drawn between the podocyte slit diaphragm and other sites of cell-cell contact, including adherens junctions and post-synaptic dendritic spines [47]–[50]. A number of important analogies between podocytes and neurons suggest that they have several common structural and functional features [47]. For example, both have a well developed and dense actin cytoskeleton and cellular processes. An important cytoskeletal component of podocytes, synaptopodin, has also been shown to have important functions in synaptic transmission at dendritic spines and another important dendritic spine component, dendrin, was recently shown to also be a component of the slit diaphragm [47], [51]. The submembranous region of both dendritic spines and slit diaphragms consists of detergent insoluble protein complexes that are believed to be organizers of signaling complexes. In the podocyte (and other cell types) these have been referred to as “lipid rafts,” whereas a well studied analogous structure at the dendritic spines is termed the post-synaptic density (PSD) [50]. Interestingly, several of the proteins encoded by these mRNAs are dendritic spine proteins that have been extensively characterized to have essential functions during synaptic development and function. In all, 15 of the enriched mRNAs have been shown by several studies to be components of dendritic spines generally, or specifically in the post-synaptic densities (PSD): MYH10, DBN1, IQGAP1, PPP1R9A, PPP1R9B, SPNB2, MYO6, ACTN4, ACTN1, MYH9, MYO18A, MYO1B, MYO5A, GSN, and PLEC1 [52]. Additionally, the podocyte foot processes have important parallels with other dynamic actin-based cellular projections such as lamellipodia, filopodia, podosomes, and pseudopodia [42], [53]. A number of the proteins encoded by these mRNAs have been shown to be components of or participate in formation of these cellular compartments: MYO6, IQGAP1, T-plastin, MYH9, actinin, filamin, MYO18A, drebrin, and MYO1B [36], [54]–[57]. Thus, it may prove the case that some of the other less characterized proteins encoded by the co-purified mRNAs will also be found to be important components of microfilament networks in these subcellular structures and processes.

The unexpected finding that the identified transcripts were not associated with WT1(+KTS) prompted us to attempt to uncover a plausible explanation to account for the identification of this subset of mRNAs encoding a remarkable functionally interrelated set of proteins. The identification of actin and EF1α in the co-eluted fractions provides some insight into the means by which these mRNAs were enriched and may further provide mechanistic insights into the cell biological implications of their co-enrichment. Most simply, it appears that semi-soluble cytoskeletal protein complexes were trapped on the beads during washes, but eluted when this material was more forcefully separated from the beads using a spin column. This possibility would be consistent with a previous study showing that components of the actin cytoskeleton, including actinins and filamins can be physically entrapped on magnetic microbeads [58]. However, one explanation that might then be suggested is that the mRNAs we identified were all co-purified in association with the encoded proteins. However, this not likely the case as Western blots comparing input and purified eluate samples probing for Myh10, Dbn1, and Iqgap1 resulted in strong signals for these proteins in the respective input samples. By contrast, there was little or no detectable level of these proteins eluate samples (data not shown). Since only the mRNAs and not the corresponding proteins were enriched in the purified eluate samples, these results suggest that our mRNAs were not merely identified by means of trapping or pulling down protein components of the cytoskeleton. Although it is possible that the co-purification of these mRNAs may have occurred due to unanticipated binding of a different RNA binding protein to the column, we find this less likely. Our mass spectrometry data are not consistent with this possibility and the lack of a common binding motif among these transcripts suggests they are bound by more than a single RNA binding protein.

The presence of EF1α might provide additional insight into how these mRNAs may have been identified and suggest the interesting hypothesis that they share a common subcellular localization and undergo localized translation. EF1α has been shown to associate with the cytoskeleton through actin binding sites. It has further been proposed that EF1α associated with a subpopulation of actin filaments to which mRNA is localized [59]. The same authors further proposed that EF1α may bind localized mRNAs to actin filaments at sites where they are anchored. It is worth noting in the context of analogies between the podocyte slit diaphragm and neuronal dendrites that numerous mRNAs that encode dendritic proteins are themselves localized to dendritic spines where they appear to be dynamically translated at their sites of expression [60] Just as a number of the proteins encoded by these mRNA are localized to dendrites, it is also noteworthy that 15 of the enriched mRNAs themselves have been shown to localize to dendritic spines, the post-synaptic density, or (in one case) at the post-synaptic membrane of the neuromuscular junction: *Ppp1r9b*, *Dbn1*, *Myh9*, *Plec1*, *Spna2*, *Spnb2*, *Actn4*, *Actn1*, *Gsn*, *Nes, Pls3, Utrn*, *Dst*, *Myo1b*, *Shrm* [61]–[69]. Since 7 of these proteins (as well as Myh10) have been shown to be localized to podocyte foot processes, these collective observations raise an interesting speculation that a number of the RNA transcripts we identified may similarly be localized at the slit diaphragm. It might be the case that these transcripts undergo dynamic translation at sites of expression in foot processes in response to extracellular cues and signaling pathways. However, while this is an intriguing possibility, further more complex cell biological studies would clearly be needed to provide direct evidence of this hypothesis.

Despite the potential implications of this work towards the identification of a putative protein interaction network, we were unfortunately unable to provide further insight into the hypothesized roles of WT1(+KTS) as an RNA binding protein. While we were unable to identify true WT1-associated mRNAs in this work, a number of technical factors may have interfered with the identification of such mRNAs. For example, the significant enrichment of mRNAs that were present in the semi-soluble protein fractions that we isolated may have masked the identification of such transcripts. Thus, further studies are needed to further investigate the proposed post-transcriptional functions of WT1 in podocytes as well as other cell types in which it is expressed.

In conclusion, while the findings we obtained were not successful in identifying novel WT1(+KTS) RNA targets, the mRNAs identified through microarray analysis revealed a surprising set of gene products with remarkably interrelated functions in maintenance of the actin cytoskeleton, including several with known important roles in podocytes as well as neuronal dendrites. Further investigation of several of the less well characterized proteins encoded by these mRNAs may uncover previously unknown components and pathways that are essential for the function of these specialized cell types.

## Materials and Methods

### Plasmids

The expression vector used to facilitate production of C-terminal FF-ZZ tagged WT1(+KTS) in stably transfected MPC-5 mouse podocyte cells has previously been described in detail [27].

### Cell Culture

MPC-5 cells were propagated at 33^°^C in RPMI 1640 with 25 mM HEPES cell culture medium (Gibco) supplemented with 10% fetal bovine serum, 10 U/ml interferon gamma (Boehringer), 100 units/ml penicillin, 100 mg/ml streptomycin, 10 µg/ml insulin-transferrin-sodium selenite (Roche). The MPC-5 cells were allowed to differentiate by withdrawal of interferon gamma from the culture medium and transferred from 33^°^C to 37^°^C for 14 days [29]. Cells were transfected using Lipofectamine 2000 (Invitrogen) according to the manufacturer's instructions and stable selection was carried out using 0.5 µg/ml of G418 (Gibco).

### Preparation of cell extracts and quantification of protein content

MPC-5 cell extracts were prepared as previously described in [27]. The total amount of protein in extract preparations was quantified using the Bradford protein assay (Bio-Rad). All extracts were prepared from differentiated MPC-5 cells.

### Ribonucleoprotein immunoprecipitation (RNP-IP) and RNA precipitation

Immunoprecipitation using IgG resin (Amersham) binding to the ZZ epitope tag of recombinant WT1 protein has previously been described, with the exception that RNase A was not added with the third wash in step 23 ([27]; steps 18 through 28). After TEV protease-cleavage and elution from the IgG beads, the eluted ribonucleoprotein fraction was directly subjected to RNA precipitation using TRIzol reagent (Invitrogen) per manufacturer's instructions. This RNA precipitation was carried out concomitantly with that of the respective input sample. RNA samples were quantified and confirmed for purity using the NanoDrop spectrophotometer and Agilent Bioanalyzer, respectively.

### mRNA amplification, labeling, and hybridization to Illumina mouse Sentrix-6 microarrays

650 ng of purified total RNA from four replicates each of input control extract or immunoprecipitated sample were single-round amplified and biotin-labeled using the Ambion Message-Amp II-Biotin Enhanced RNA amplification kit (Applied Biosystems) according to manufacturer's instructions. Amplified cRNA samples were quantified and confirmed for purity using the NanoDrop spectrophotometer and Agilent Bioanalyzer, respectively. After fragmentation, the biotin-labeled antisense cRNAs were hybridized to Illumina Sentrix-6 Mouse Expression Beadchip (Illumina) containing 46,657 probe sets. Raw data from the microarray analysis has been deposited in the Gene Expression Omnibus (GEO) database at the National Center for Biotechnology Information (NCBI) website under accession code GSE16942.

### Microarray Analysis

The results were first filtered for probesets that were detected above an expression threshold (“MPC-5 transcriptome”). Spotfire 9.0 was used to select probesets among the experimental hybridization samples with a detection value of≥0.95 (p-value 0.05) in at least three of the four experimental replicates. Of the 46,657 probesets on the CHIP, 15,094 probesets passed this detectable expression filter, which roughly corresponded to a signal intensity of approximately 100 at the lower end. After log_2_ transformation, a two way mixed model ANOVA across all four pairs was used to calculate false discovery rates (FDR) using the resulting p-values according to the Benjamini-Hochberg method as implemented in Partek Genomics Suite v. 6.3 [70]. Using a 15% FDR cutoff we identified a total of1764 probesets that demonstrated a difference in average expression between to experimental and control sets (including both positive or negative differences). The complete list of these probesets is presented in Table S1.

### Gene Ontogeny (GO) Analysis

Of the 60 genes that passed the 15% FDR and the two-fold cutoff, 55 either had pre-assigned Human Gene Nomencltaure Committee (HCGN) approved gene symbols in the data output, or the gene symbol could be manually assigned by searching BLAT in the UCSC genome browser using the probe sequence. These 55 symbols were input as a gene list into the DAVID Bioinformatics Database and assigned DAVID ID's. The three riken clones that passed the 15% FDR and two-fold cutoff were excluded from this analysis. Of the over 15,000 genes that passed the expression threshold, less than half could be identified in the DAVID Bioinformatics Database and assigned DAVID ID's using the available accession numbers in the results files. Therefore, we instead used the entire mouse genome list in DAVID as the background set for the comparison with the 55 genes from the enriched dataset. GO analysis was completed analyzing all molecular functions, cellular components, and biological processes [31], [32].

### Antibodies and Immunoblotting

Immunoblotting was performed as described in [71]. Antibodies used were anti-flag M2 mouse monoclonal antibody (Sigma; 1∶1,000 dilution), Anti-anti-β-actin clone AC-15 (Sigma; 1∶1000 dilution), anti-elongation factor tu clone sc-21758 (Santa Cruz; 1∶50 dilution).

### Real-time qRT-PCR

One microgram of total RNA was used to generate cDNA with the High-Capacity cDNA Reverse Transcription Kit (Applied Biosystems). Analysis was performed in triplicate using TaqMan Gene Expression Assays for Myh10 (Assay ID: Mm00805131_m1), Flnb (Assay ID: Mm01311723_m1), Dbn1 (Assay ID: Mm00517314_m1), Svil (Assay ID: Mm01233200_m1), Spnb2 (Assay ID: Mm00486365_m1), Actn4 (Assay ID: Mm00502489_m1), Nes (Assay ID: Mm00450205_m1), Iqgap1 (Assay ID: Mm01313746_m1), Spna2 (Assay ID: Mm01326617_m1), Dst (Assay ID: Mm01227142_m1), Actn1 (Mm01304398_m1), Tmod3 (Assay ID: Mm00497523_m1), Gapdh (Assay ID: Mm99999915_g1) normalized to Ubiquitin C (Assay ID: Mm01201237_m1) (Applied Biosystems). The ddCt method of relative quantification was used on a 7500 Fast Real-Time PCR machine with SDS software (Applied Biosystems).

## Supporting Information

Table S1 Complete list of all probesets that passed the expression filter
(0.29 MB XLS)
